# A High-Precision Micro-Roll Forming Facility for Fuel Cell Metal Bipolar Plate Production

**DOI:** 10.3390/mi16010091

**Published:** 2025-01-14

**Authors:** Matthias Weiss, Peng Zhang, Michael Pereira

**Affiliations:** 1 Institute for Frontier Materials, Deakin University, 75 Pigdons Rd., Waurn Ponds, Geelong, VIC 3216, Australia; peng.neo.zhang@deakin.edu.au; 2 School of Engineering, Deakin University, 75 Pigdons Rd., Waurn Ponds, Geelong, VIC 3216, Australia; michael.pereira@deakin.edu.au

**Keywords:** micro-roll forming, ultra-thin metal sheet, bipolar plate, tool manufacturing

## Abstract

The metal bipolar plate is a critical component of the hydrogen fuel cell stack used in proton exchange membrane fuel cells. Bipolar plates must have high accuracy micro-channels with a high aspect ratio (*AR*) between the channel depth and the half periodic width to achieve optimal cell performance. Conventional forming methods, such as micro-stamping, hydroforming, and rubber pad forming, cannot achieve these high ARs given that in these processes, material deformation is dominated by stretch deformation. In micro-roll forming the major deformation mode is bending, and this enables production of channels with higher *ARs* than is currently possible. However, micro-roll forming uses multiple sets of forming roll stands to form the part and this leads to technological challenges related to tool alignment and roll tool precision that must be overcome before widespread application can be achieved. This study presents a new methodology to achieve tight tool tolerances when producing micro-roll tooling by utilizing wire-EDM and micro-turning techniques. This is combined with a new micro-roll former design that enables high-precision tool alignment across multiple roll stations. Proof of concept is provided through micro-roll forming trials performed on ultra-thin titanium sheets that show that the proposed technology can achieve tight dimensional tolerances in the sub-millimeter scale that suits bipolar plate applications.

## 1. Introduction

Recent advances in battery and fuel cell technology have led to growing vehicle electrification. The hydrogen fuel cell electric vehicle has a high energy storage density and can be refueled faster than the battery electric counterparts; this provides opportunities for heavy-duty and long-range transport applications [1]. An important component of the fuel cell system is the bipolar plate (BPP) which provides the flow field path for the reactant gases and fluids inside the fuel cell stack [2] and conducts electricity. This flow path is typically achieved in the bipolar plates which use many small sub-millimeter size flow channels to evenly distribute the flow of the anode, cathode, and coolant fluids over a large number of individual cells within the fuel cell stack. There can be several hundred cells within a fuel cell stack. Therefore, approximately 300–400 million bipolar plates are needed annually to equip 500,000 vehicles, as each vehicle contains hundreds of bipolar plates [3,4,5].

Metal bipolar plates can be mass manufactured at high volumes and therefore have gained increasing attention in recent years [6]. There are several well-known methods to produce bipolar plates from thin metal sheets, such as microstamping [7,8], hydroforming [9,10], and rubber pad forming [11]. Roller embossing has been introduced recently [12,13] and promises potential advantages in terms of continuous production and reduced tooling and manufacturing facility costs. The performance of a metal bipolar plate increases with the aspect ratio, *AR*, of the micro flow channels, which is the ratio of the flow channel depth to its half periodic width. The main limitation of current forming methods is that they rely on the stretch forming of material, which limits the *AR* that can be achieved before necking or fracture occurs. The aspect ratio reported for single-stage microstamping is in the range 0.4–0.5 and increases to 0.6–0.7 for multistage microstamping [14]. Hydroforming has been shown to enable a 20% improvement compared to conventional single-stage microstamping [15], while for gear forming [16] higher *ARs*, of up to 1.0, were observed and related to material deformation being limited to 4-point bending, which prevents the stretching and necking of material. However, the *AR* in gear forming is still limited due to tool geometry restrictions. Micro-roll forming [17,18] enables the forming of microchannels from a continuously fed coil. The major deformation mode in micro-roll forming is incremental bending and this enables the manufacture of a high *AR* and reduces material thinning. However, the process requires a large number of forming stations that contain high-accuracy roll tooling and that must be carefully aligned with each other. For example, it has been shown in previous work [17] that tool shape and alignment inaccuracies within a magnitude of less than 50 µm can result in undesired material thinning or coining of material. Tool alignment in a conventional roll-forming mill is generally achieved using dial gauges [19], piano wires/strings, and optical or laser-assisted devices [20]. However, these solutions cannot achieve the roll alignment tolerances required for micro-roll forming.

Several micro-machining methods such as wire-cut electrical discharge machining, often referred to as wire-EDM [21], and micro-end-milling [22] have been successfully applied to produce high-precision micro-stamping dies. Die-sinking EDM is another technique [23,24] where an electrode is cut by wire-EDM or end-milling to create a negative shape of the desired tool profile. The electrode is then used to erode the final die profile by electrical discharge. These methods are applicable to microstamping dies [12,13,16] but cannot be applied to produce micro-forming rolls, which require the cutting of grooves around the circumference of the roll. Rotary wire-EDM could achieve the micro-profile shape on a cylindrical part but would require a rotary table that is submersible in the dielectric fluid which would increase process costs and complexity. Micro-turning used for turning micro-grooves on a lathe represents a more cost-effective approach but is prone to process chattering which can lead to low turning precision [25].

This study aimed to develop a new micro-roll forming facility that overcomes tool misalignment issues that currently prevent the widespread implementation of the process for metal bipolar plate manufacture. This was combined with a new approach for producing high-precision micro-forming rolls. The approach uses wire-EDM to cut a high-precision negative tool that is subsequently used in a micro-turning process to cut high-precision micro-channel profiles into steel cylinders to produce micro-roll forming tools. To prove the concept, micro-roll forming trials were performed on ultra-thin titanium sheets. It was shown that the new high-precision micro-roll forming concept enables the manufacture of micro-channels that meet industrial shape tolerance demands relevant to fuel cell bipolar plate manufacture and the high *AR* needed for fuel cell performance.

## 2. Experimental Methodology and Setup

### 2.1. The Designed Part and Roll Tool Profile Geometry

**Figure 1:**
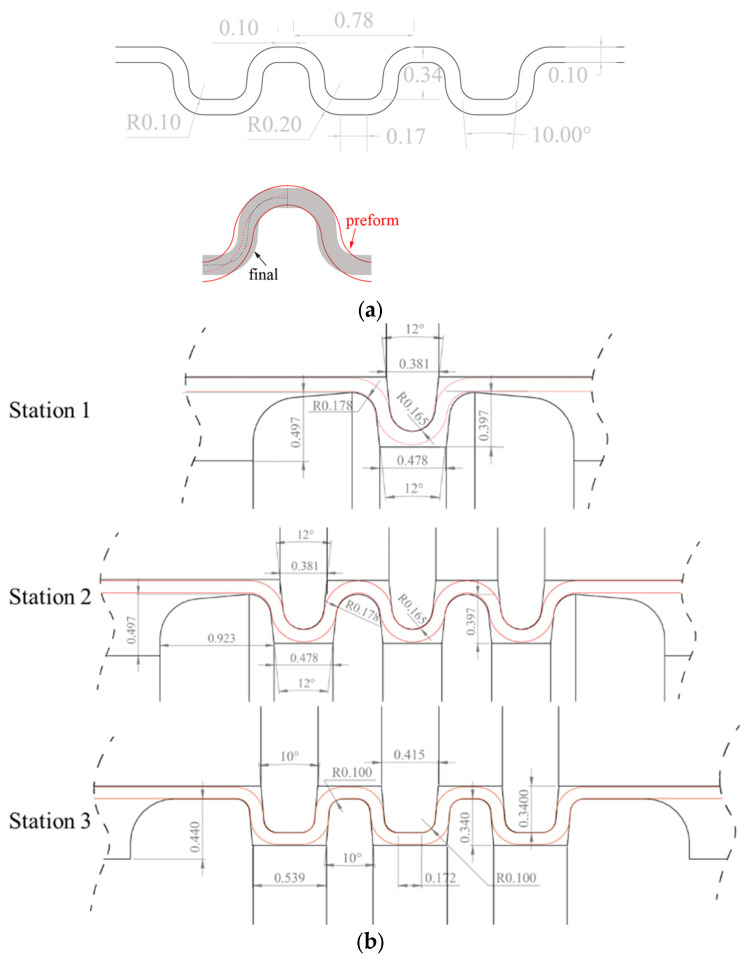
(**a**) Final geometry of the micro-channels to be manufactured by micro-roll forming (units in mm) (**b**) Forming roll geometry of preform-stations 1 and 2 and sizing station 3 (units: mm), the sheet profile at each station is outlined in red.

The target profile is a three-channel shape (Figure 1a) with a depth of 0.34 mm and a half period length of 0.44 mm. This represents a typical parallel straight shape relevant to the microchannels in industrially relevant metal bipolar plates, with an *AR* of 0.77. The profile is formed in three roll-forming passes using the micro-roll forming approach presented by [17], where a preform is first produced (stations 1 and 2), followed by the material being pushed into the corner radii in a final sizing step (station 3), as shown in Figure 1b. In station 1, the middle channel is formed first, and the two side channels are formed in station 2. This enables material to flow freely and reduces the likelihood of transverse stresses and stretching of the material. In station 3 the material is pushed into the tight corners of the final profile shape design. Forming the corrugations in this way reduces material deformation to nearly pure bending and enables the direct correlation of the critical forming zones of the formed corrugation profile with the shape accuracy of the micro-rollers.

### 2.2. Design and Manufacture of the High-Precision Roll Former

**Figure 2:**
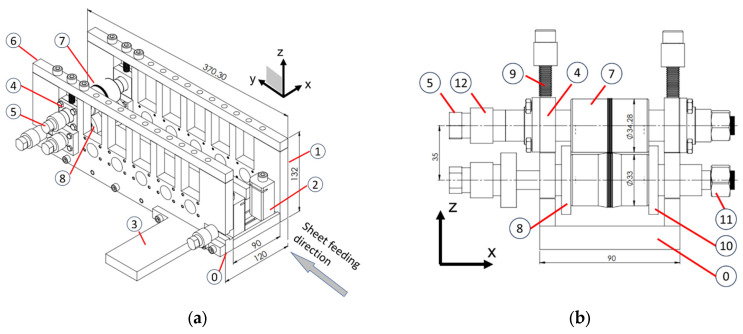
(**a**) Schematic of the high-precision micro-roll former with (**b**) the last station installed. Dimensions are not to scale (units: mm). Circled numbers indicate the following parts: 0—base, 1—side plate, 2—feeder station, 3—motor/handle drive location, 4—bottom/top bearing housing, 5—shaft, 6—top block, 7—top roll, 8—bottom roll, 9—gap adjusting screws, 10—locking spacers, 11—locking nut, and 12—pulleys to connect the timing belt.

**Figure 3:**
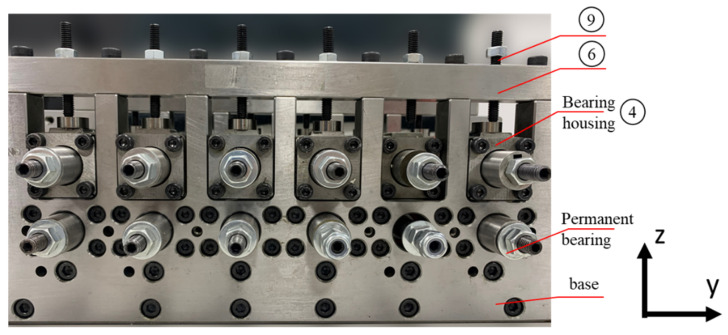
Photograph of the left side of the high-precision micro-roll former, showing the permanent bearings which are used to position the bottom roll shafts. The circled numbers are in accordance with Figure 2.

**Figure 4:**
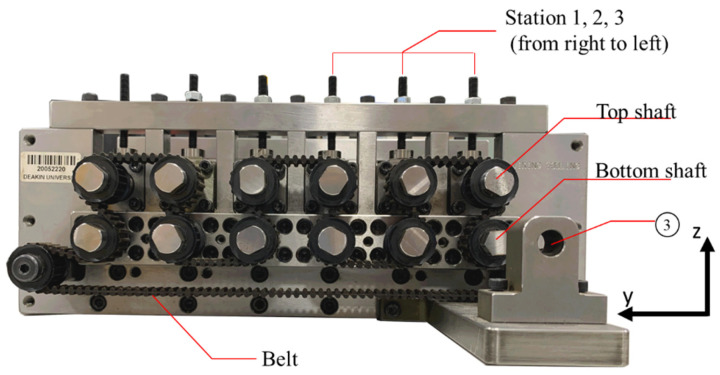
Photograph of the right side of the high-precision micro-roll former, showing how the timing belt connects the bottom and top roll shafts (view from the opposite side compared to Figure 3).

The high-precision micro-roll former concept is shown in the drawings in Figure 2 and photos are provided in Figure 3 and Figure 4. As shown, the micro-roll former includes a base with a U-channel shape that is manufactured from hardened low-alloyed tool steel to provide high rigidity. The base (⓪ in Figure 2a) is milled out from one entire block within a tolerance of 0.001 mm and has two side plates ① connected on either side using dowel pins. The bottom roll shafts are held by six pairs of permanent bearings that are fixed in the base (Figure 3). This provides a reference plane on the left side and ensures that the bottom rolls in all forming stations are aligned horizontally to each other within a tolerance of 5% of the 0.1 mm sheet thickness, i.e., 0.005 mm.

The bearing housings of the top rolls are positioned in the machined slots of the base sidewalls and held with a top block (⑥ in Figure 3) that is bolted to the side wall. The horizontal alignment of the top and bottom rolls in each individual station is achieved with locking spacers that are machined to give a tolerance of 0.005 mm (⑩ in Figure 2b).

The vertical position of the top roll can be further fine adjusted with shims that are placed between the top bearing housing (④) and the permanent bearing housing that holds the bottom roll shaft. The top bearing housing is pushed onto the bottom bearing housing with the adjusting screws (⑨). All rolls are driven by a crank handle (placed at ③, as shown in Figure 4) which is attached to a timing belt. All forming rolls have the same diameter to give a speed ratio of 1:1 between the top and bottom rolls. The station distance between each of the forming stations is 47 mm.

### 2.3. Procedure for High-Precision Roll Tool Manufacture

**Figure 5:**
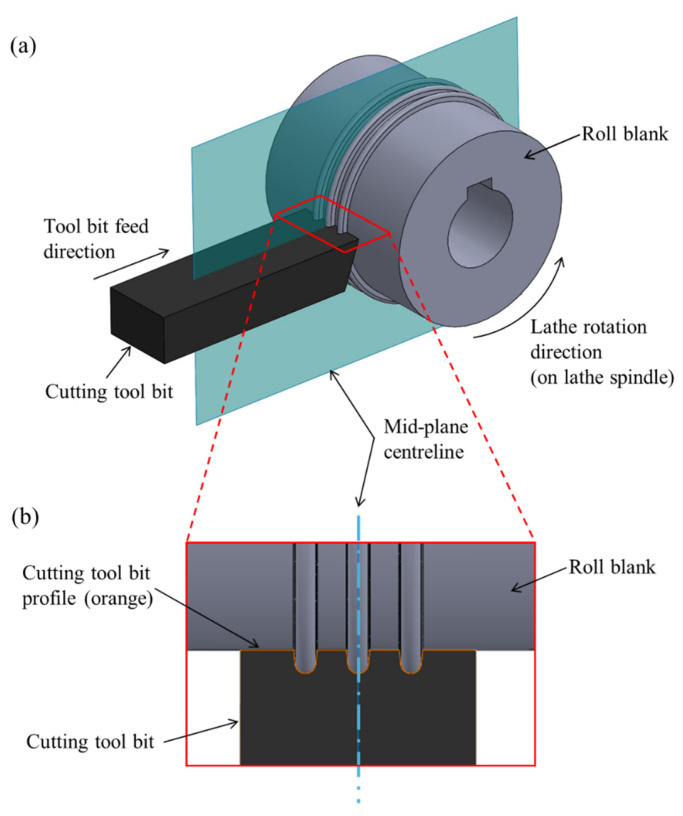
(**a**) Schematic of the roll turning process to achieve the desired corrugation profile, and (**b**) a detailed view, showing the cutting tool bit profile. Schematics not to scale.

The corrugated tool profile of the forming rolls as shown in Figure 1 must be turned on a lathe machine because of its axisymmetric shape. To achieve this, a cylindrical roll blank without the corrugated profile was first turned on a lathe using conventional methods. Subsequently, to achieve the desired high-precision corrugated profile, a high-speed hardened tool-steel cutting tool bit was used to machine the tool profile on the cylindrical roll blank on a lathe, as shown in Figure 5a. The tool profile for the cutting tool was produced by wire-EDM cutting, with the pattern of the cutting edge being the negative shape of the desired top or bottom roll profile. The protrusions and the grooves on the rolls are therefore the grooves and the protrusions on the tool bit. For the wire-EDM process, a wire diameter of 0.25 mm was used for producing the tool bit profile for stations 1 and 2, while a wire diameter of 0.1 mm was used for station 3 due to the smaller corner radii required (see Figure 1b).

An example of the tool profile geometry is shown in Figure 5b. This geometry was first analyzed using optical profilometry to check the accuracy (see Section 2.5) and then used to turn the corrugation profile on the roll blanks to manufacture the forming rolls. The symmetrical line (mid-plane shown in Figure 5) to be cut on the roll blank was aligned with the tool bit center to ensure an accurate positioning of the corrugations on the rolls. The best position accuracy achieved with this method was approximately ±0.05 mm, which is insufficient for the micro-roll former tooling. To achieve the high-precision roll alignment required (approximately ±0.005 mm), subsequent steps were conducted as described in Section 2.4.

A turning speed of 70 rpm was used in combination with water-soluble cutting lubricant for cooling and to reduce friction. To improve the surface finish at the end of the turning process, polishing paste with 3 µm diamond particles (Diapro-Struers, Ballerup, Denmark) was used. The polishing paste was smeared on a microfiber towel, which was then applied to the roll tools which were spinning on the lathe.

### 2.4. Procedure for High-Precision Roll Alignment

**Figure 6:**
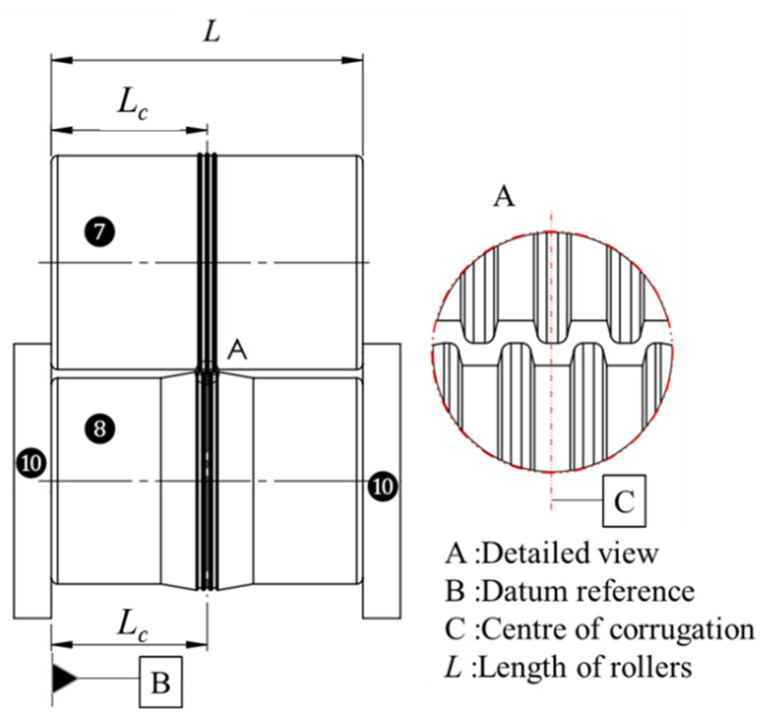
Schematic of the ideal alignment between top and bottom micro-channels (the roll gap is schematically exaggerated). Labels: 7-top roll, 8-bottom roll, and 10-locking spacers.

As described in Section 2.2, the special roll former hardware design enables a vertical and horizontal roll tool alignment in each and between individual stations that is theoretically within 0.005 mm. Due to this design, the tool position in the roll former and the alignment between the top and bottom rolls (Figure 6) depend on the dimensional accuracy of the top and bottom rolls. Ideal alignment means that the centerline of the top roll corrugation coincides with the centerline of the bottom corrugation (as shown by centerline C in Figure 6). For this, the distance *L_c_* from the corrugation centerline to the left reference plane (Datum B in Figure 6) must be 0.005 mm, for both the top and bottom rolls. From the point of manufacturing, it is easier to achieve high precision for the total width of the cylindrical roll rather than to ensure a tight tolerance for the horizontal position of the center corrugation over the width of the roll.

**Figure 7:**
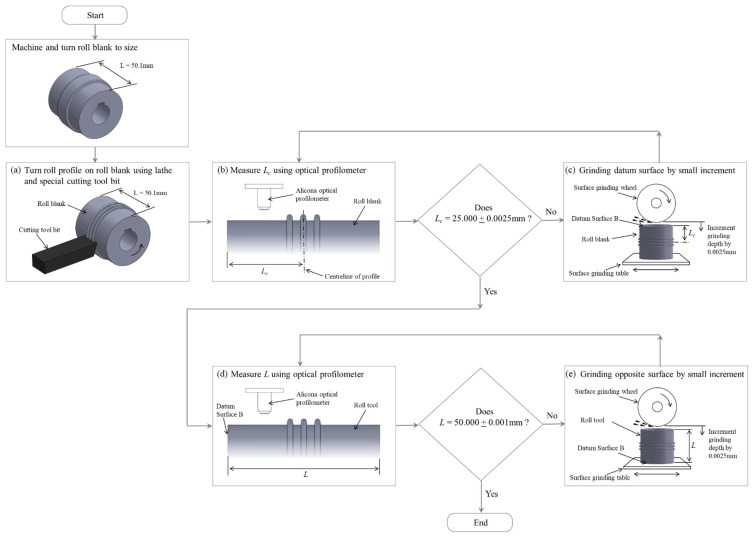
Flow chart of the steps of machining and measurement to achieve high-precision roll alignment.

A high precision center positioning of the metal sheet in the roll former was achieved with three approaches: (I) At the start of forming, the sheet was feed into the first forming station with a guide station (② in Figure 2). (II) The minimum length of the precut sheet was more than 6-times (300 mm) the station distance (47 mm); this ensured that when the center length of the strip was formed the sheet was guided by all roll stations. (III) The special procedure for high-precision roll alignment (Section 2.4) ensured that the center corrugation of the strip was aligned and followed a straight line through the roll former. To achieve the required high precision for (III), a special procedure was applied, which is summarized by the flowchart in Figure 7. First, all rolls were pre-cut from a cylindrical rod with an initial length *L* = 50.1 mm, which is 0.1 mm longer than the required roll width. These pre-cut roll blanks were then turned on a lathe (Figure 7a), using the procedure described in Section 2.3. After turning, an optical profilometer (Alicona InfiniteFocus G5, Raaba, Austria) was used to measure the distance *L_c_* of the corrugation center to the datum surface B (Figure 7b). A surface grinding machine was then used to grind down the critical roll surface B in multiple steps (Figure 7c). For this, the roll was positioned vertically with an electromagnetic fixture, and a precision grinding wheel was used to achieve a fine surface finish. Between each of the surface grinding steps, additional optical profilometry measurements were performed to measure $L_{c}$. Using this procedure, a tolerance of 0.0025 mm was achieved for the corrugation center position distance to the left reference plane (Datum B) for both the top and the bottom roll. Subsequently, the opposite roll surfaces were ground (Figure 7d) to achieve the total width *L* = 2$L_{c}=$ 50.000 ± 0.003 mm (Figure 6) for both the top and the bottom roll. With the center corrugation of both the top and the bottom rolls now aligned within 0.0025 mm to the left reference plane, and the top roll position in relation to the bottom roll being fixed with locking spacers (⑩ in Figure 6), the overall horizontal tool alignment is within 0.005 mm.

### 2.5. Analysis of Tool Profile Shape Accuracy

An optical profilometer (Alicona InfiniteFocus G5) was used to measure the accuracy of the tool bits and roll profiles. The vertical and lateral resolution was set for image acquisition as 5 µm and 7 µm, respectively, to achieve a high resolution combined with the shortest possible scanning time.

**Figure 8:**
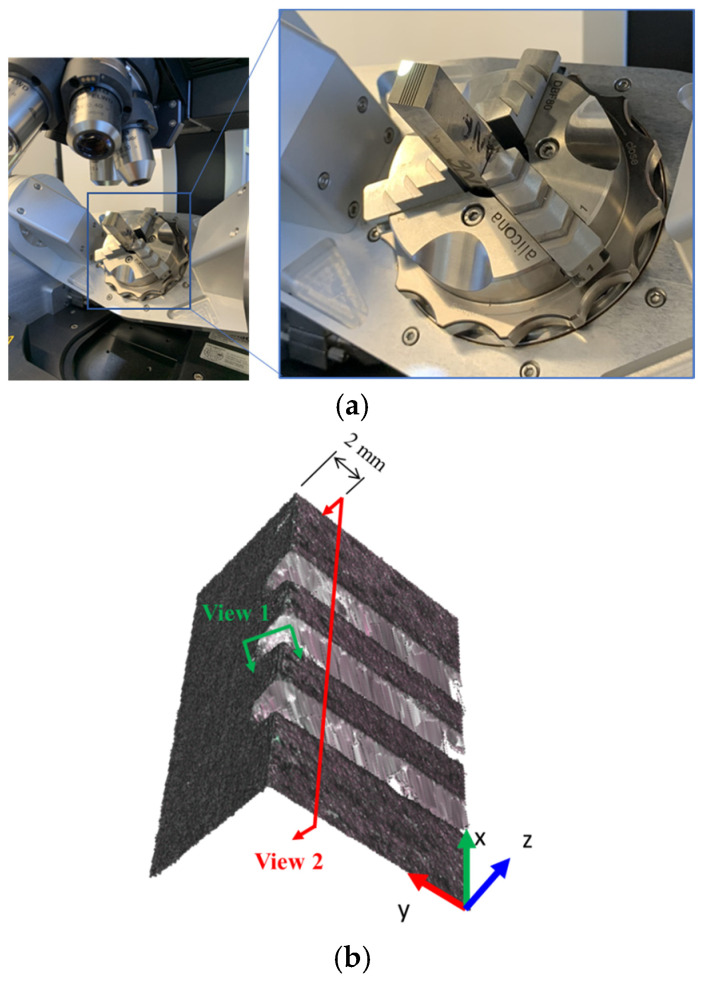
(**a**) Tool bit position during the scan and (**b**) 2D cross-section views measured from the 3D surface profilometry scanning data.

The tool bit was mounted on the specimen stage at a 45° inclined angle (see Figure 8a). Using the Alicona InfiniteFocus measurement suite (v5.3.2), two section-views (Figure 8b) were constructed to measure the tool bit before and after it was used for cutting the rollers. View-1 was aligned with the center of the male profile of the tool bit parallel to the y–z plane (Figure 8b) while View-2 was parallel to the x–z plane and 2 mm offset from the corner edge of the tool bit. The Section Results presents the results regarding the three tool bit conditions before and after cutting.

**Figure 9:**
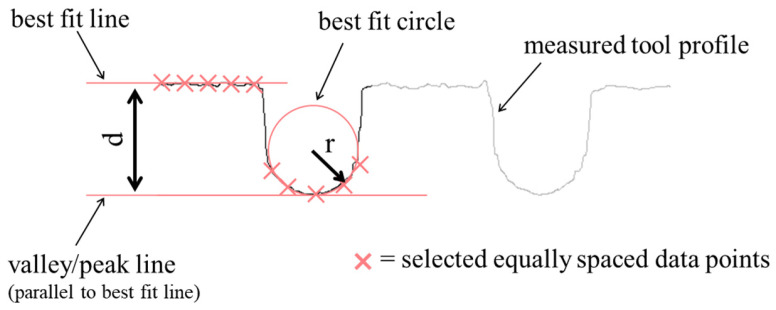
Example measurement method to determine the accuracy of the roll tool profile radius (r) and profile depth (d).

3D surface profile scanning of the roll tools was also conducted with the Alicona, using the same settings as used for scanning the tool bits. The measurement focused on the 2D cross-section profile with four equally spaced measurements performed over the 360° circumference. The profiles were then analyzed in the Alicona InfiniteFocus measurement software to determine the valley depth, d, and radii, r, of the manufactured roll tool profiles (Figure 9). The measured radii and depths were then compared to the designed (ideal) profile shape of Figure 1b. To measure the radius, r, five equally spaced points along the measured radius were selected and a circle of best fit was measured. For the flat (straight line) regions of the profile, again five equally spaced points were selected, and a line of best fit was generated. These lines were then used for the measurement of the valley depth, d, as shown in Figure 9. Finally, the shape error of the radii and the depths of the produced tool shape (Figure 1b) when compared to the designed roll tool profile were determined.

### 2.6. Roll Forming Trials

#### 2.6.1. Material

**Figure 10:**
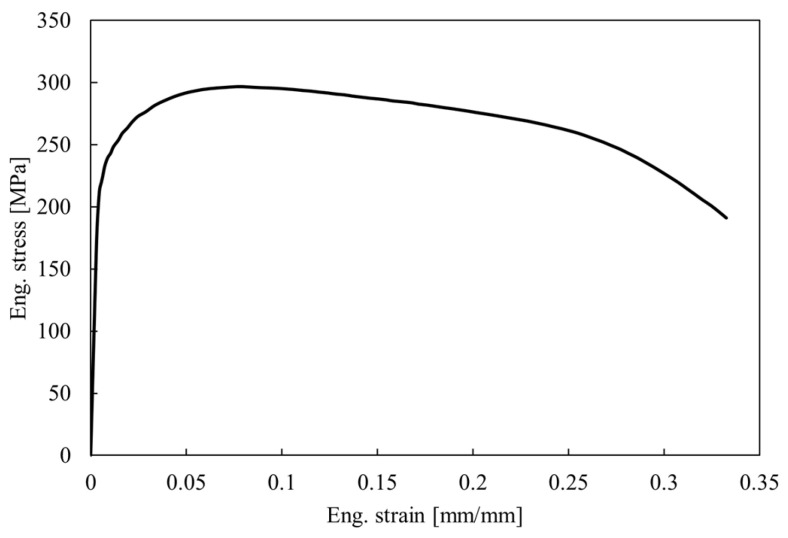
Representative engineering stress–strain curve of a CP-Ti tensile sample tested transverse to the rolling direction.

The material used for the roll forming test was a commercial pure titanium (CP-Ti, grade 2) of 0.1 mm thickness (supplier: Ti-Shop, London, UK). Titanium sample sheets of 50 mm (*W*) × 300 mm (*L*) were produced with a guillotine and tensile test specimens [26] cut transverse to the rolling direction with wire-EDM cutting. A typical tensile test curve is shown in Figure 10 and the average yield and tensile strength were 226 MPa and 297 MPa, respectively.

The roll tools were manufactured from AISI D2 tool steel and cleaned with ethanol; no additional heat treatment was applied.

#### 2.6.2. Roll Forming Test Conditions

The high-precision micro-roll former schematically shown in Figure 2 was used to conduct the roll-forming trials. In all cases, the titanium sheet rolling direction was aligned with the feed-in direction of the roll former. The specimens were fed into the micro-roll former by hand and the rolls were driven by the manual crank handle. Formed sheets were removed after stations 1, 2, and 3, and the part profile shape and material thinning were examined after each roll station. All roll-forming trials were conducted without any lubrication.

#### 2.6.3. Analysis of the Sheet Profile Shape

To examine the cross-section of the formed channels, a segment of 10 mm was cut perpendicular to the channel direction. The specimen was mounted in resin and mechanically polished and then examined with an Olympus DP71 microscope (Olympus, Tokyo, Japan). The microscope images were then analyzed with ImageJ software (1.52v) [27] to determine the thickness of the sheet. Given that in previous work a high level of material anisotropy was reported [14], care was taken to align the CP-Ti sheet rolling direction with the sheet movement direction in the roll former, i.e., the cross-section of the formed profile was oriented perpendicular to the CP-Ti sheet rolling direction/sheet movement in the roll former.

**Figure 11:**
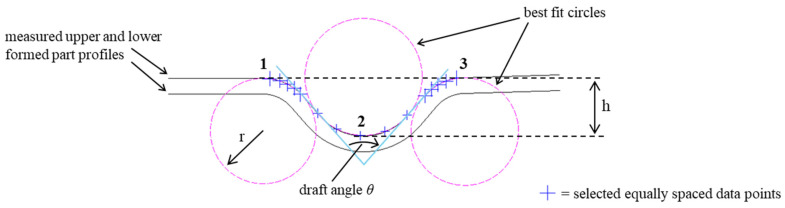
Measurement method applied to determine the radius (r), draft angle (θ), and height (h) of the formed parts.

The draft angle *θ*, shown in Figure 11, gives a measure for springback with a higher forming depth resulting in a smaller draft angle indicating less springback. In the formed sheet, the angle is measured by best fitting the side wall of the section cut with straight lines in AutoCAD. For this, the cross-section images of the sheet formed in station 1 and station 2 were analyzed to identify the upper and the lower sheet surface profiles. Using these profiles, the method for quantifying the tool geometry presented in Section 2.5 was applied to determine the geometry of the formed parts. For this, the circles that provide the best fit to the top and bottom radii of the formed part were created, and then the tangential lines of these circles were used to identify the side wall of the parts (Figure 11). The angle between the tangential lines for the neighboring sidewalls was considered to be the draft angle *θ*. This fitting method applies only to stations 1 and 2. For station 3, the formed sheet profile had a short and nearly vertical sidewall which did not allow achieving a good fit with circles to identify tangential lines. This prevented the identification of the sidewalls and the analysis of the draft angle *θ* for this condition. For station 3, therefore, only the profile height (depth) *h* was determined as the distance between the bottommost node of the bottom circle (location 2) to the line that connects the topmost points of the top circles at location 1 and location 3, see Figure 11.

## 3. Results

### 3.1. Profiles of the Tool Bit

**Figure 12:**
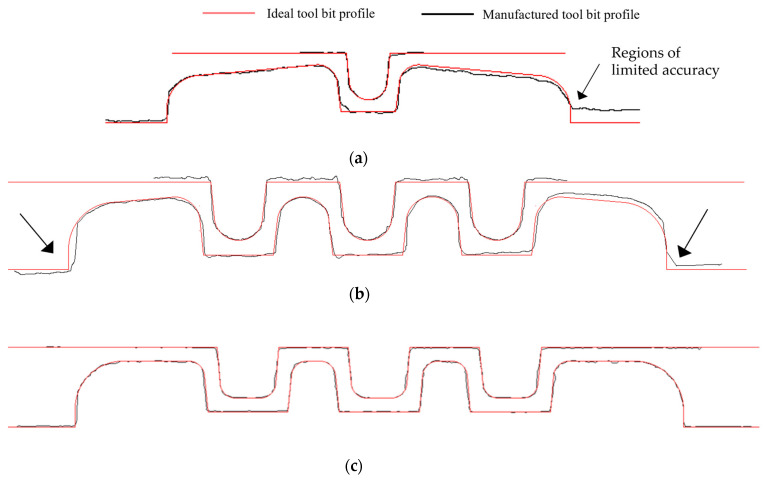
Tool bit profile cross section measured with an Alicona profilometer. The desired profile is indicated in red while the measured profile is shown in black. (**a**) Roll station 1. (**b**) Roll station 2. (**c**) Roll station 3.

The 2D cross-section profiles of the tool bit measured with the Alicona are overlayed with the ideal profiles in Figure 12. The result indicates that the tool bit obtained after wire cutting had good dimensional accuracy. Only in the outer left and right regions of the bottom rollers (stations 1 and 2) were some regions of limited accuracy observed.

### 3.2. Profile Shape and Surface Roughness of the Produced Forming Rolls

**Figure 13:**
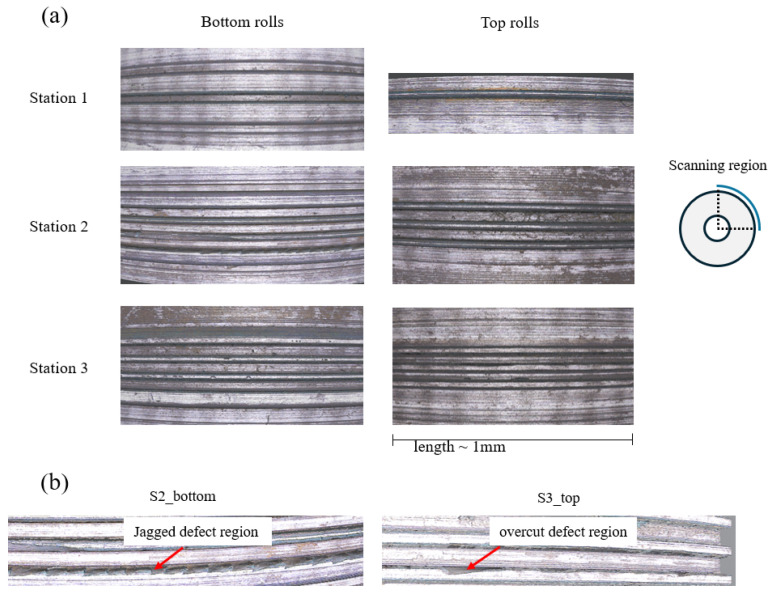
Roll profile geometry analysis. (**a**) Good regions for top and bottom rolls of stations 1–3. (**b**) Defect regions observed in the bottom and the top roll of station 2 and 3, respectively.

The roll surfaces produced after the turning process were examined over the full roll circumference with the Alicona profilometer. While most of the roll surfaces were of high accuracy (Figure 13a), several roll areas showed some manufacturing defects (Figure 13b).

For example, there was a jagged defect region in the bottom roll of forming station 2 (Figure 13b). This is likely due to chattering. The bottom rolls have a larger contact area compared to the top rolls during turning, and, therefore, it is likely that, here, a higher chattering effect occurred. The top rolls of forming stations 1 and 2 did not show any obvious surface defects. In some areas around the circumference of the top roll of forming station 3 there was some excessive material removal near the channel corner radius; this may be related to operational errors.

**Figure 14:**
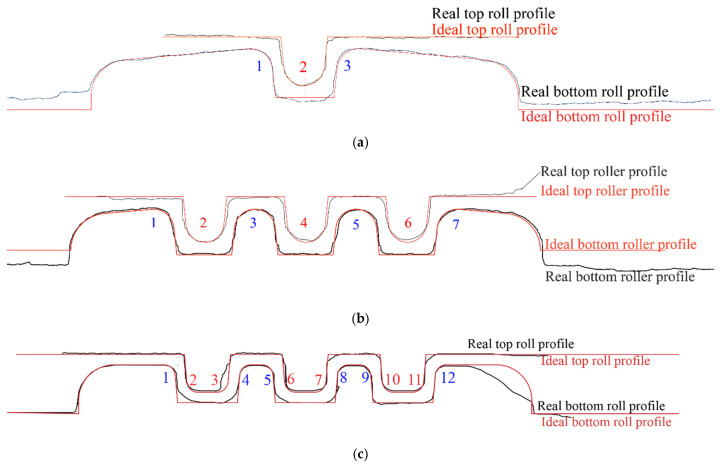
Comparison between the ideal and the measured micro-roll tool profiles: (**a**) Station 1. (**b**) Station 2. (**c**) Station 3.

Figure 14 compares the ideal and the real roll profile taken from the “good” roll regions. The forming radii of all top rolls agree well with the ideal shape. There is a clear shape error in the bottom forming roll of station 1. This error is in the form of a higher bottom roll channel depth compared to the design.

Note that in the roll forming process investigated here, material deformation is governed by the top roll dimensions and the corner radii of the bottom rolls; i.e., for this analysis the tool dimensions of the top roll (for example S1-2) and the radii of the bottom rolls (for example S1-1, S1-3) are mostly important for forming the corrugation shape. In stations 1 and 2 the critical top roll radii are indicated in red, while for the bottom they are indicated in blue. In roll station 3 all forming rolls have a left and a right profile radius, which in this study were separately numbered. Again, red indicates critical profile radii in the top roll, while blue stands for the critical bottom roll radii.

**Figure 15:**
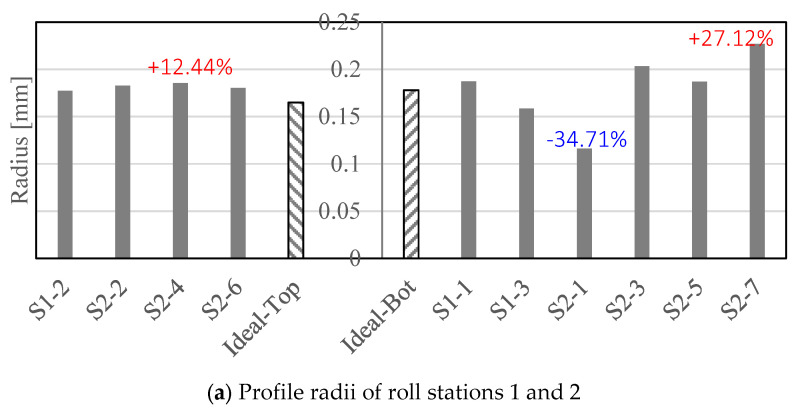

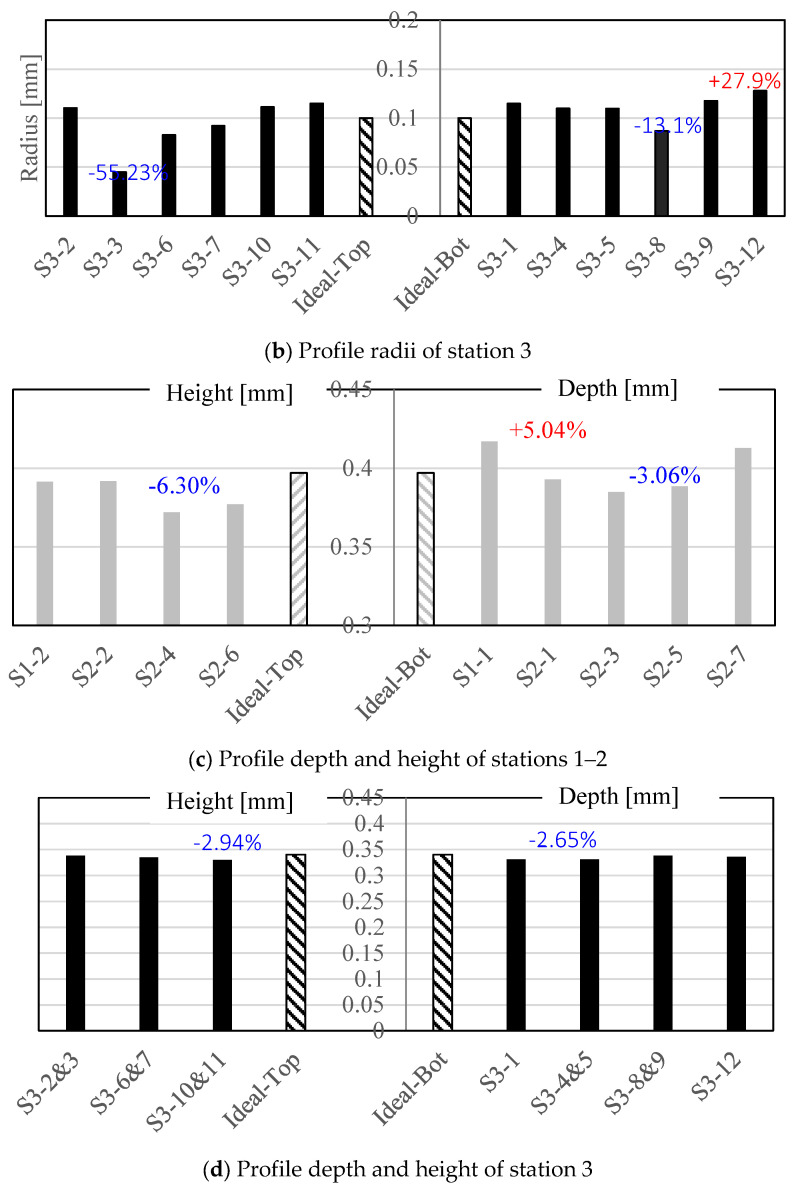
(**a**) Radius of the roll profile in stations 1 and 2, (**b**) radius of the roll profile in station 3, (**c**) height and depth of the roll profile in stations 1 and 2, (**d**) height and depth of the roll profile in stations 3. Maximum and minimum percentage errors of the ideal shape are labelled.

The measured tool profile radii are compared to the ideal (designed) radii in Figure 15 where the dimensions of the top rolls of stations 1 and 2 are shown on the left of Figure 15a and for the bottom rolls on the right. The ideal profile radius of S2-1 and S2-7 is 0.178 mm. However, the manufactured S2-1 radius was approximately 34% lower while for S2-7 it was 27% higher compared to the ideal profile radius. A significant overcut of 55% can be observed in S3-3 (Figure 15b) of the top roll which conforms to the visual observation of overcutting reported in Figure 13b.

In all roll stations on the far right and left ends of the rollers, there were some major profile errors. These are likely due to the insufficient wire-cutting accuracy achieved in these regions in the cutting tool bit as previously shown in Figure 12. However, these roll profile regions are located outside of the forming zone; i.e., the affected parts of the forming rolls do not form the metal sheet, and it is therefore unlikely that this shape error will affect the forming outcome.

For all roll stations the produced heights (depths) of both the top and bottom rolls were in accordance with the ideal shape (see Figure 15c,d), with the dimensional error being less than 7%.

**Figure 16:**
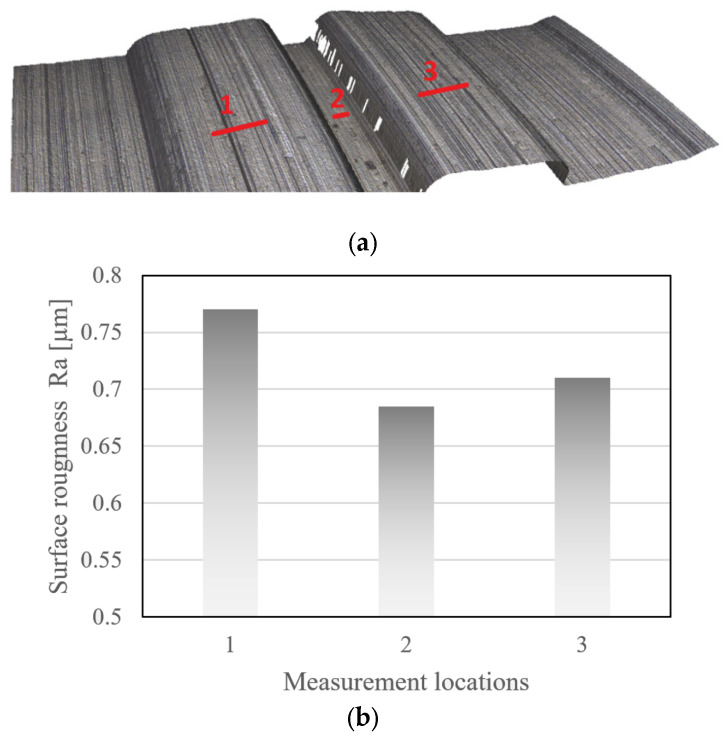
Surface roughness, *Ra*, measured for three locations on the bottom micro-roller of station 1. (**a**) Locations and path directions used for measuring the surface roughness Ra. (**b**) Surface roughness measurement values in µm for locations 1–3.

The surface roughness Ra of the roll tools was measured with the Alicona profilometer on the bottom roller of station 1; this is representative for the surface quality achieved with the cutting procedure for all micro-rollers. The surface roughness was analyzed in the three locations shown in Figure 16a) over a 10 µm line width and path lengths of 0.5 mm in locations 1 and 3, and of 0.25 mm in location 2. The Ra values shown in Figure 16b are close to those observed in previous work [13].

### 3.3. Micro-Roll Forming Trials

#### 3.3.1. Comparison of the Formed Cross-Section Shape with the Ideal Shape

**Figure 17:**
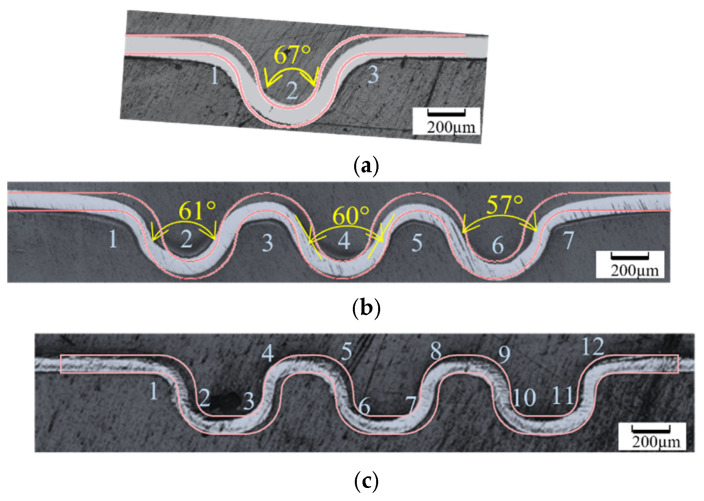
Cross-section after micro-roll forming. The profiles in orange represent the ideal shape. The draft angle is annotated in yellow, and the locations of the radius measurement are marked with white numbers. (**a**) 2D sheet profile after roll station 1. (**b**) 2D sheet profile after roll station 2. (**c**) 2D sheet profile after roll station 3.

The formed cross-section shape is visually compared with the ideal shape in Figure 17. For this, cross-section images of the formed titanium sheet determined with optical microscopy are overlayed with the ideal profile shape for both the top and the bottom sheet surface.

It becomes clear that in station 1 the formed draft angle is significantly higher than the designed draft angle of 12°. The draft angle in the center corrugation is reduced from 67° in station 1 to 60° in station 2 when the sheet is being re-rolled. In station 3 the draft angle was not measured but Figure 17c suggests a good fit between the ideal and the formed profile shape. Note, that after both forming stations 1 and 2, there was no warping of the sheet. The outer sections of the strip beyond profile radii 1 and 3 (station 1) and profile radii 1 and 7 (station 2) move slightly up. This is due to springback in the profile radii 1, 3 and 1, 7 in stations 1 and 2, respectively, and is not the result of warping.

The formed profile radii and heights are compared with the ideal profile shapes for each of the three roll forming stations and are summarized in Table 1 and Table 2. To facilitate the comparison between the formed part shape and the cross-section shape of the forming rolls the numbering of the critical radii in Table 1 was done in the same way as for the roll profile radii shown in Figure 14 and Figure 15. For example, the radius location 1 of the part cross section shape analyzed after forming station 1 (Table 1) corresponds to the roll profile radius S1-1 in Figure 15a. In Table 1, it becomes clear that in several part locations, the formed micro-channel profile radius was not accurately formed. After forming station 1, the formed profile radius 1 is approximately 25% higher than the ideal radius while the other two radii have shape errors of approximately 10% or less. The profile radii formed in station 2 are within 14% of the ideal profile radii while in station 3, four out of 12 profile radii show shape deviations of 20% and higher. The highest shape deviation is observed in profile radius location 3 where the formed profile radius is more than 122% higher than the ideal profile shape.

The channel depth, *h*, of the formed cross sections measured after the three individual forming stations are given in Table 2. While in forming stations 1 and 2 all channel depths were under-formed, in station 3 the depth of all channels was between 16 and 18% higher compared to the ideal profile.

#### 3.3.2. Material Thinning

**Figure 18:**
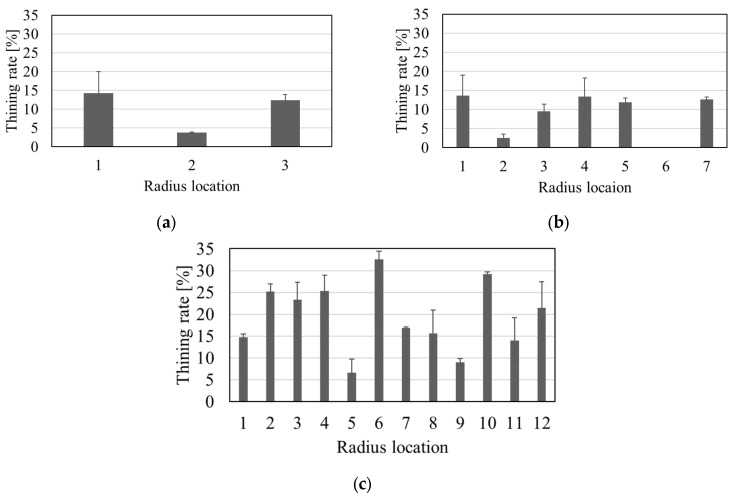
Material thinning in the part profile radius, (**a**) after roll station 1, (**b**) after roll station 2, (**c**) after station 3. The radius locations are visually shown in Figure 18.

Material thinning was measured using the pixel method [17]. The thinning at the radius location was measured in three proximity locations, and the averaged values are shown (in percentage) in Figure 18. Stations 1 and 2 show a maximum thinning of 14.25% and 13.31%, respectively, while there is significantly higher thinning in station 3, with the maximum thinning of 32.5% found in radius 6 which is positioned in the center of the middle groove.

## 4. Discussion

### 4.1. The Effect of the Cutting Bid Shape Accuracy on the Micro-Roller Profile and the Formed Micro-Channel Profile Radius

Figure 12 suggests that the wire-cutting process applied in this study can produce an accurate 2D micro-profile on the tool bid. Some shape error was observed but limited to the outer right and left tool bid regions that are not relevant to the forming process. When using the produced tool bid to turn the micro-roller profiles, a high-profile accuracy was achieved for the top rolls of stations 1 and 2 with the profile shape error being less than 12%. The bottom roll of station 2 shows major shape deviation in profile radius locations 1 and 7 (see Figure 14b and Figure 15a, on the right). These locations conform to the shape inaccuracy observed in the far left and right tool bid zones in Figure 12b. This confirms that shape errors in the cutting tool bid directly influence the profile accuracy in the turned roll tools. However, while the tool bids for station 3 had the highest accuracy (Figure 12c), profile radius 12 of the produced bottom roll of station 3 showed a deviation of more than 27% from the ideal shape (Figure 13b, right) while location 3 of the top roll had a profile radius that was more than 50% lower compared to that designed (Figure 13b, left). Similar to this, while the tool bid used for cutting the bottom roll profile of station 1 conformed well with that designed, the micro-profile shape of the bottom roll after turning had a micro-channel depth that was higher than that of the ideal shape, see Figure 14a.

Figure 13b suggests that jagging and overcutting occurred during the micro-roll turning process and this may explain why even with a nearly perfect tool bid cross-section, some shape error was still introduced into the micro-roll surface profiles.

The effect of the micro-roll surface profile on the part quality appears to depend on the type and location of the shape error. As shown in Table 2, the shape error in profile radii 1 and 7 after forming the titanium foil in station 2 is less than 13%. In contrast to this the roll profile radius shape error in the bottom roll was 53 and 27%, respectively, in the roll areas that formed these two sheet locations. The shape error in the bottom micro-roll tool is positioned in the left and the right outer roll regions, and this is where the material is less restricted during the forming process. This may explain why the roll tool shape error only has a minor effect on the formed micro-channel profile shape accuracy. In contrast to this the shape error due to overcutting observed in profile radius S3-3 of the top roll of station 3 (Figure 14c and Figure 15b) leads to a formed profile radius that is 120% larger than that designed. Profile radius 3 is positioned in the center of the sheet where the forming severity is the highest and the material is fully restricted by the neighboring micro-channels. This suggests that the effect of the roll tool shape accuracy on the component quality depends on the forming zone. Note, that even though the shape accuracy of the top and the bottom micro-rollers of stations 3 was within 13%, the profile radii formed in locations 2, 6, 7, 10, and 11 in station 3 were between 55 and 81% higher compared to the ideal shape. This could be related to the very tight profile radius of 0.1 mm that was formed here which would have led to high forming forces and tool deflection, possibly preventing the sheet material from being fully pushed into the tight corner radius profile.

The results of this study therefore suggest that the effect of the micro-roll tool profile accuracy depends on the forming region and the severity of forming. Only for some cases, such as for example profile radius S3-3, was a direct link between tool accuracy (Figure 15b) and the formed profile quality (Table 1) established.

### 4.2. The Effect of Springback

The final roll-formed part profile shape is clearly affected by springback. While the channel heights and depths of the roll tooling only had an error of −6.3% and +5.12% in roll stations 1 and 2 (Figure 15c), the forming depth in station 1 was under-formed by more than 20%, see S1-2 in Table 2. In station 2 the forming depth was between 11 and 13% lower than that designed. This suggests that the lower forming depth achieved is to a major extent due to springback and not a result of the shape error of the forming rolls. Figure 16 shows that after forming stations 1 and 2 the draft angle of the formed micro-channels was more than five times higher than that designed; this confirms that there was a high level of springback after the material had been formed in stations 1 and 2.

After forming in station 3 the micro-channel profile depth appears to be between 16 and 18% higher compared to the designed profile while the error in the roll tool profile accuracy was much lower and under 3%. Again, this can be related to springback. In station 3 the round bottom channel profile that comes from station 2 is formed into a trapezoidal shape with a flat bottom. So, like the part profile shape after forming stations 1 and 2, also in forming station 3, the profile depth accuracy mostly depends on the level of springback and to a lesser extent on the roll profile accuracy.

### 4.3. Tool Alignment and Material Thinning

The new roll former design was aimed to resolve the tool alignment issues observed in previous work [17] that led to excessive material thinning. Based on the results shown in Figure 18, it can be concluded that in roll forming stations 1 and 2 the roll tooling has been successfully aligned given that on both sides of the roll formed channel profile there is an even thinning distribution; i.e., in station 1 (Figure 18a) profile radii 1 and 3 show similar thinning while in station 2 (Figure 18b) the same applies to profile radii 1-3 and 5-7. In forming station 3 the distribution of material thinning still appears slightly uneven. The maximum material thinning observed in station 3 is approximately 32.45% which is very similar to [17] where the maximum material thinning was 31% in the final forming (sizing) station. However, it must be noted that in [17] an *AR* of 0.6 was roll formed while in the current study the *AR* is 0.77 which is more than 20% higher. This suggests that in the current micro-roll former set-up the max material thinning has been reduced, and this may indicate that material thinning was more balanced due to the improved alignment of the roll tooling.

### 4.4. Future Applications

**Figure 19:**
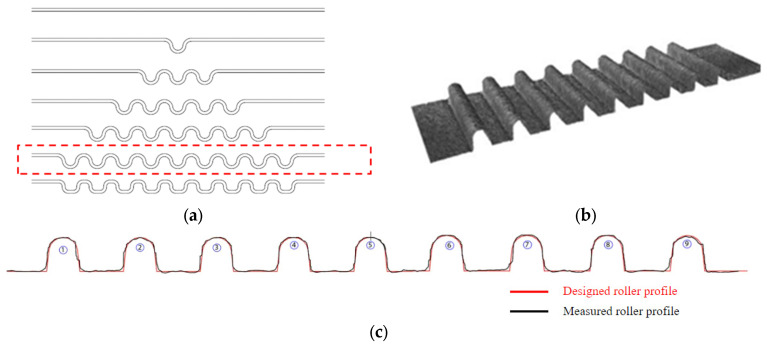
(**a**) Theoretical roll forming bending sequence for producing 9 micro-channels. (**b**) Scanned top roll tool surface profile. (**c**) Comparison between the designed and measured 2D tool profile.

The current study is limited to the production and testing of high precision micro-rollers with tool profiles of three micro-channels and lower; this only has a small industrial significance. To prove that the new method can successfully produce micro-forming rollers with a higher number of micro-channel profiles, the presented cutting method was applied to produce nine micro-channels on a top micro-roller tool and the Alicona profilometer was used to analyze the surface profile accuracy achieved. The micro-roll forming sequence required to theoretically produce these nine micro-channels is presented in Figure 19a; i.e., five forming stations would be required to produce nine micro-channels with two micro-channels being formed in each subsequent forming station. Based on this, it can be concluded that the production of 50 micro-channels would require a minimum of 26 roll stations; this is a common number of forming stations in conventional roll forming. While the 3D surface scan of the produced top roll is shown in Figure 19b the 2D cross section is compared to that of the designed profile in Figure 19c. The result suggests that the presented cutting method is applicable for the high precision production of multiple micro-channel roll tool profiles relevant to industrial and high-volume micro-channel production.

## 5. Conclusions

The paper presents a new micro-manufacturing facility for the micro-roll forming of channel profiles relevant to metal bipolar plate production for fuel cells. The work is a continuation of a previous study that showed that micro-roll forming presents a low-cost alternative to the conventional micro-stamping process for high volume bipolar plate production. The previous work highlighted that significant improvements to the produced micro-roll tool accuracy and tool alignment are necessary to achieve the required product quality. This paper attempts to address this need in two ways. First, by the development of a new micro-roll forming facility that includes advanced solutions for high precision tool alignment. Second, with the development of a low-cost micro-roller manufacturing process that applies wire cutting to first produce a steel cutting tool, followed by a turning process where the cutting tool is used to produce the high precision roll surface profile. This study presents these new solutions and applies surface profilometry and optical microscopy to investigate the tool profile accuracy that is achieved and how this affects the accuracy of a part profile micro-roll formed from titanium foil. The following conclusions can be made.

- Wire cutting allows the production of the steel cutting tool with high accuracy. However, when applied to the roll turning process, jagging and overcutting lead to some shape errors on the produced micro-roll profile. Future work needs to investigate the application of lubrication and coolant fluids to overcome this issue.
- Outside the regions where jagging and overcutting occurred, the micro-roll tool profile was formed with an accuracy that was between −13% and 12% of the designed shape.
- In most cases the product accuracy achieved when roll forming the titanium foil was not directly linked to the roll profile shape accuracy. On the contrary this study showed that springback accounted for most of the shape errors observed.
- The new micro-roll former design led to a clear improvement in tool alignment that manifested itself by a more evenly distributed and lower level of material thinning in the micro-roll formed titanium profile compared to previous studies.

## Figures and Tables

**Table 1:** Radius of the formed channel of the sheet after each station. Radius locations marked with white numbers in Figure 18.

	Radius Location	Ideal Radius [mm]	Measured Radius [mm]	Error [%]
station 1	1	0.28	0.35	25.00
	2	0.17	0.19	10.86
	3	0.28	0.29	3.22
station 2	1	0.28	0.31	10.28
	2	0.17	0.18	7.02
	3	0.28	0.24	−13.98
	4	0.17	0.18	7.36
	5	0.28	0.26	−8.47
	6	0.17	0.18	7.91
	7	0.28	0.25	−10.43
station 3	1	0.20	0.24	22.19
	2	0.10	0.16	61.63
	3	0.10	0.22	122.68
	4	0.20	0.17	−13.55
	5	0.20	0.23	14.75
	6	0.10	0.17	70.15
	7	0.10	0.18	81.56
	8	0.20	0.23	14.77
	9	0.20	0.26	30.61
	10	0.10	0.16	60.79
	11	0.10	0.16	55.86
	12	0.20	0.22	11.60

**Table 2:** Depth of the formed channel of the sheet after each station. Channel numbered from left to right.

	Channel Number	Ideal Depth [mm]	Measured Depth [mm]	Error [%]
station 1	S1-2	0.40	0.310	−21.27
station 2	S2-2 (left)	0.40	0.349	−12.72
	S2-4 (middle)	0.40	0.346	−13.51
	S2-6 (right)	0.40	0.354	−11.61
station 3	S3-2&3 (left)	0.34	0.396	16.47
	S3-6&7 (middle)	0.34	0.404	18.87
	S3-10&11 (right)	0.34	0.399	17.31

## Data Availability

Data are contained within the article.
